# Evolution and transmission landscape of the staphylococcal *msrA* gene mediating resistance to 14-membered macrolides and type B streptogramins

**DOI:** 10.3389/fmicb.2026.1815688

**Published:** 2026-04-22

**Authors:** Davit Janelidze, Saba Kobakhidze, Tinatin Elbakidze, Mamuka Kotetishvili

**Affiliations:** One Health Institute, School of Science and Technology, University of Georgia, Tbilisi, Georgia

**Keywords:** antimicrobial resistance, antimicrobial resistance genes, horizontal gene transfer, macrolide, MsrA, recombination, *Staphylococcus*, type B streptogramin

## Abstract

**Introduction:**

*Staphylococcus* species, particularly *Staphylococcus aureus*, are leading opportunistic pathogens responsible for a wide range of infections, with antimicrobial resistance—including high rates of macrolide resistance—severely limiting treatment options. The *msrA* gene encodes the ABC-F protein MsrA, which mediates inducible resistance to 14-membered macrolides and type B streptogramins. Despite its clinical and epidemiological relevance, the evolutionary forces, selective pressures, and transmission routes shaping *msrA* in staphylococci remain insufficiently understood.

**Methods:**

Six hundred and one complete staphylococcal *msrA* coding sequences (CDSs) were retrieved from GenBank. Evolutionary analyses of *msrA* included nucleotide diversity (*π*), selection metrics (*d_N_-d_S_*, π_a_/π_s_, Tajima’s D, Fu’s Fs, FUBAR, MEME, and aBSREL), and conservation mapping using DnaSP in relation to MsrA functional domains (UniProt P23212). Linkage disequilibrium (LD) was assessed using ZnS, Za, ZZ, and Wall’s statistics. Recombination and transmission pathways were inferred using GARD, RDP4-embedded algorithms, SplitsTree network analysis, and the PHI test.

**Results:**

Forty-one *msrA* allelic variants were determined, with five predominant alleles accounting for approximately 90% of CDSs; allele 19 was almost exclusive to *S. aureus*. Nucleotide diversity was moderate (*π* ≈ 0.039–0.042), and strong purifying selection predominated (π_a_/π_s_ ≈ 0.169; *d_N_-d_S_* = −0.138 ± 0.016; strongly negative Fu’s Fs), with only four codons showing evidence of episodic positive selection. Three highly conserved regions were identified, mainly overlapping the inter-domain linker and the second nucleotide-binding domain across MsrA. Moderate-to-high LD with minimal decay indicated the persistence of only a limited number of successful allelic variants. Predominant *msrA* alleles were largely plasmid-associated. Recombination analyses revealed frequent interspecies transfer within *Staphylococcus*, with *S. aureus* acting as a central donor to *Staphylococcus chromogenes* and *Staphylococcus saprophyticus*, as well as rare intergeneric transfers involving *Citrobacter*, *Enterococcus*, *Corynebacterium*, and *Pseudomonas*.

**Conclusion:**

These findings support a dual evolutionary strategy for *msrA*: strong purifying selection preserves its essential ribosomal-protection function, while plasmid-mediated dissemination promotes the spread of fit alleles. *S. aureus* appears to be a key reservoir and vector, facilitating both interspecies and intergeneric transmission. Clinically, this underscores the need for surveillance of plasmid-borne *msrA* and targeted control of *S. aureus* reservoirs to limit resistance to macrolides and type B streptogramins.

## 1 Introduction

*Staphylococcus* species, particularly *Staphylococcus aureus*, are among the most important opportunistic pathogens, causing a broad spectrum of infections ranging from superficial skin and soft tissue infections to severe invasive diseases such as bacteremia, pneumonia, endocarditis, and device-related infections (Tong et al., 2015; Cheung et al., 2021). In 2019, *S. aureus* was associated with more than one million deaths worldwide, highlighting its substantial global public health burden (Ikuta et al., 2022). The emergence and dissemination of antimicrobial resistance (AMR) in staphylococci—most notably methicillin-resistant *S. aureus* (MRSA) and coagulase-negative staphylococci (CoNS)—have severely limited treatment options and complicated clinical management (An et al., 2024). In parallel, the growing prevalence of macrolide-resistant staphylococci poses an additional challenge, contributing to increased infection rates and adverse outcomes, particularly in healthcare-associated settings where *S. aureus* remains a leading cause of morbidity and mortality (An et al., 2024). A recent comprehensive systematic review and meta-analysis revealed alarmingly high global pooled prevalences of resistance to erythromycin, clarithromycin, and azithromycin among *Staphylococcus* spp., estimated at 57.3, 52.6, and 57.9%, respectively (Navidifar et al., 2025). Collectively, these findings underscore a troubling global pattern of macrolide resistance and highlight the urgent need for deeper investigation into the molecular mechanisms driving this phenomenon in these organisms.

In staphylococci, resistance to macrolides is predominantly mediated by *erm*-encoded 23S rRNA methyltransferases (ermA, ermB, ermC), which confer the MLS B phenotype through target modification. In contrast, the *msrA* gene, which encodes an ABC-F ribosomal protection protein, represents the most common non-*erm* mechanism conferring resistance to 14- and 15-membered macrolides and streptogramin B antibiotics (Ross et al., 1990; Reynolds et al., 2003). The relative prevalence of *erm* and *msrA* varies substantially among staphylococcal populations depending on geographic region, species (e.g., *S. aureus* vs. coagulase-negative staphylococci), methicillin resistance status, and clinical setting (Miklasińska-Majdanik, 2021; El Mammery et al., 2023). Overall, *erm* genes—particularly *ermC*—have been studied far more extensively than *msrA*, although the latter may appear to be prominent in specific epidemiological contexts or resistance phenotypes. Notably, although the MsrA protein encoded by *msrA* was initially proposed to function as a classical efflux pump, accumulating structural and functional evidence has demonstrated that it confers resistance through direct ribosomal protection. MsrA binds to the ribosome and, in an ATP-dependent manner, dislodge bound antibiotics from the nascent peptide exit tunnel (NPET), thereby restoring translation (Sharkey et al., 2016; Wilson, 2016).

The *msrA* gene is frequently plasmid-borne, facilitating horizontal gene transfer (HGT) via conjugation, transduction, or mobilization within and between staphylococcal species (Feßler et al., 2018). Evidence of interspecies—and occasionally intergeneric—dissemination has been reported, contributing to the broad host range of *msrA* across *Staphylococcus* spp. and, sporadically, beyond this genus (e.g., *Enterococcus*, *Pseudomonas*, and *Corynebacterium*) (Ojo et al., 2006). This pronounced genetic mobility, coupled with sustained selective pressure from macrolide use in clinical, veterinary, and agricultural settings, accelerates the global spread of macrolide resistance and complicates efforts to control antimicrobial resistance (AMR). Despite the clear clinical relevance of this antimicrobial resistance determinant, however, the evolutionary dynamics of staphylococcal *msrA* loci, and their specific transmission trajectories operating within *Staphylococcus* remain poorly understood.

Here, we present a comprehensive evolutionary analysis of the major *msrA* allelic variants in staphylococci. By integrating nucleotide diversity metrics, selection and conservation analyses, linkage disequilibrium profiling, and multiple recombination-detection frameworks, we show that *msrA* evolution is dominated by strong purifying selection acting on a limited number of highly successful allelic lineages. The functional regions were found to differ in their variability, with the inter-domain linker and nucleotide-binding domain 2 exhibiting stronger functional constraints across the *msrA* gene. Moreover, our findings reveal extensive plasmid-associated dissemination, as well as specific interspecies and intergeneric recombination events, positioning *S. aureus* as a central hub within the *msrA* donor–recipient network. It appears that, through intergeneric recombination, staphylococci have been responsible for the transmission of *msrA* not only to *Enterococcus*, *Corynebacterium*, and *Pseudomonas*, but also to *Citrobacter*. Collectively, these findings provide novel and important evolutionary and epidemiological insights into the persistence, mobility, and dissemination of staphylococcal *msrA* loci.

## 2 Materials and methods

### 2.1 *msrA* DNA sequences

A total of 601 coding sequences (CDSs) of the 1,467-bp *msrA* gene (Supplementary Table S1) were selected and analyzed in the GenBank database of the National Center for Biotechnology Information (NCBI)^1^. Only the *msrA* CDSs without ambiguous nucleotides and/or low-quality sequence signals were included in the study. These DNA sequences originated from a diverse range of bacterial species and genera, spanning a broad taxonomic spectrum.

### 2.2 DNA sequence homology analysis

The *msrA* CDSs were retrieved from GenBank, utilizing the Basic Local Alignment Search Tool (BLAST) with the megaBLAST algorithm. The *Staphylococcus* reference *msrA* CDS (accession no. X52085.1), encoding an ABC-F subfamily ribosomal protection protein and curated in the Comprehensive Antibiotic Resistance Database (CARD^2^), was used as the query to retrieve homologous DNA sequences from the GenBank nucleotide database. The BLAST analysis was performed using the following default general and scoring parameters: word size = 28; expected threshold = 0.05; maximum matches in a query range = 0; gap costs = linear; match/mismatch scores = 1/−2; extension = 2. Regions characterized by low compositional complexity were masked. Poor-quality *msrA* entries, particularly those with ambiguous bases, were also eliminated. The BLAST searches using the reference *msrA* CDS (X52085.1) retained only sequences with 100% query coverage and ≥85% DNA identity for inclusion in the downstream analyses.

### 2.3 DNA polymorphism and selection analyses

To analyze the evolutionary patterns of the gene, the *msrA* CDSs were aligned using ClustalX v2.1 (Larkin et al., 2007). We used MEGA v12 (Kumar et al., 2024) to estimate nucleotide substitution probabilities based on Maximum Likelihood (ML) (Tamura and Nei, 1993) and Maximum Composite Likelihood (MCL) methods (Tamura et al., 2004) across the aligned *msrA* allelic types. The transition/transversion bias was assessed under the Kimura two-parameter model (Kimura, 1980). Additionally, separate transition/transversion rate ratios for purines (*k₁*) and pyrimidines (*k₂*) were estimated under a model allowing unequal base frequencies. The overall transition/transversion bias (*R*) for this gene was calculated as described previously (Tamura et al., 2004).

Further evolutionary analyses were conducted using DnaSP v6.0 (Rozas et al., 2017), which included, but were not limited to, determining invariable sites, singleton sites, parsimony-informative sites, allelic diversity, and nucleotide diversity (*π*) within the *msrA* gene. To assess selective pressures on *msrA*, we applied several methods across different software platforms: The difference between nonsynonymous and synonymous substitutions (*d_N_-d_S_*) was estimated using the Nei–Gojobori method (Nei and Gojobori, 1986) with Jukes–Cantor correction in MEGA12, with variance assessed via 1,000 bootstrap replicates; deviations from neutrality were evaluated using the codon-based Z-test of selection and Fisher’s Exact Test implemented in MEGA12, both of which compare *d_N_* and *d_S_* substitution patterns to neutral expectations; the nucleotide diversity (π) at synonymous (π_s_) and nonsynonymous (π_a_) sites was calculated, along with Tajima’s *D* (Tajima, 1989) and Fu and Li’s statistics (Fu and Li, 1993), as implemented in DnaSP v6.0; to detect selection signatures further, we also applied the Fast Unconstrained Bayesian AppRoximation (FUBAR) method (Murrell et al., 2013), using a posterior probability threshold of 0.9 to identify pervasive positive/diversifying and negative/purifying selection; MEME (Murrell et al., 2012) was used to detect individual sites under episodic diversifying selection, and aBSREL (Smith et al., 2015), using a likelihood ratio test, was applied to identify episodic diversifying selection on specific branches of the *msrA* phylogeny. In addition, FoldX (v.5.0) (Delgado et al., 2019) was employed to evaluate the structural impact of amino acid substitutions at sites identified as being under positive diversifying selection by MEME. For each site, the corresponding point mutations were introduced into the protein structure, and changes in folding free energy (ΔΔG, kcal/mol) were calculated using the FoldX *BuildModel* function under default parameters.

Conserved regions within the *msrA* gene were determined applying the Conserved DNA Regions module of DnaSP v6.0, which uses a sliding-window analysis to detect segments enriched in invariant sites across the DNA alignment (C = 1 – S/L, where *S* is the number of segregating sites and *L* is the window length). Regions exhibiting low polymorphism and statistically significant conservation (*p* < 0.05, hypergeometric test) were determined as conserved, in accordance with integrative sequence-evolution principles (Vingron et al., 2009). In addition, we applied UniProt^3^ to define the functional architecture of MsrA, identifying its duplicated ATP-binding cassette nucleotide-binding domains and conserved inter-domain regions in this protein. Subsequently, we assessed overlapping relationships between the determined conserved regions and the nucleotide-binding domain 1 (NBD1), nucleotide-binding domain 2 (NBD2), and the inter-domain linker across the MsrA protein (UniProt accession: P23212).

A ML phylogenetic tree of *msrA* gene alleles was reconstructed using IQ-TREE v3.0.1 (Minh et al., 2020), with ModelFinder employed to identify the best-fit substitution model (Kalyaanamoorthy et al., 2017). Branch support was assessed using 1,000 nonparametric bootstrap replicates. Because no suitable external outgroup was available, the ML tree was rooted using the most divergent allele in the dataset as a putative outgroup. The resulting tree was visualized and annotated using iTOL^4^.

### 2.4 Genetic recombination and homoplasy tests

We applied DnaSP v6.0 to investigate patterns of linkage disequilibrium (LD) across the alleles of the *msrA* gene, employing the ZnS statistic (Kelly, 1997), along with Za and ZZ metrics (Rozas et al., 2001), each calculated using 10,000 coalescent simulations to generate neutral expectations. These simulations were also applied to assess non-random associations among polymorphic sites, using Wall’s *B* and *Q* summary statistics (Wall, 1999). The ZnS, Za, and ZZ statistics quantify overall and local LD by summarizing non-random associations among polymorphic sites rather than relying on fixed significance thresholds. In contrast, Wall’s *B* and *Q* evaluate the spatial distribution of mutations along the *msrA* sequence, with elevated values indicating clustering of polymorphisms rather than random placement—patterns that may reflect localized recombination suppression, selective processes, or historical demographic events. Statistical significance for all summary LD statistics was assessed through coalescent simulations under a neutral evolutionary model. In addition, pairwise LD coefficients (D, D′, and R) and the corresponding Fisher’s Exact and χ^2^ test values were calculated for all polymorphic site pairs using DnaSP v6.0.

To detect evidence of genetic recombination within *msrA’s* allelic variants, we used the Genetic Algorithm for Recombination Detection (GARD) (Kosakovsky Pond et al., 2006). Using GARD, breakpoint inference was conducted under an ML framework, incorporating a general discrete model of site-specific rate heterogeneity with four rate categories. These analyses were performed using the universal genetic code and the Normal run settings, enabling the identification of putative recombination breakpoints across the aligned DNA sequences of *msrA*.

Transmission networks of putative *msrA* donors and recipients were inferred, using a large array of the recombination detection algorithms implemented in RDP4 (v.4.101) (Martin et al., 2015): In particular, RDP (Martin and Rybicki, 2000), GENECONV (Padidam et al., 1999), BootScan (Martin et al., 2005), MaxChi (Smith, 1992), Chimaera (Posada and Crandall, 2001), SiScan (Gibbs et al., 2000), and 3Seq (Boni et al., 2007) were applied to reconstruct the *msrA* transmission networks and infer potential donor–recipient relationships; using RDP4, we determined recombination beginning and end breakpoints across the allelic types of *msrA*, and identified representative putative donors and respective putative recombinants of this gene among different bacterial species and genera included in this study. In all RDP4 analyses, we retained default permutation settings (1,000 permutations), enabled the option to disentangle overlapping recombination signals, and conducted analyses under the linear genome configuration. To ensure high confidence in recombination detection, we adopted a very stringent filtering criterion: Only the breakpoint clusters supported with ≥99% confidence and associated with Bonferroni-corrected *p*-values ≤ 0.05 were considered statistically significant.

Independent evidence for interspecies and intergeneric recombination events was also evaluated using the split decomposition method implemented in SplitsTree v6.7.0 (Bandelt and Dress, 1992; Huson and Bryant, 2006). The analysis focused on the *msrA* allelic sequences representing the donor–recombinant pairs randomly sampled from the groups previously inferred by RDP4. SplitsTree visualizations allowed to further validate potential interspecies and intergeneric recombination events as network parallelograms—distinct patterns indicative of conflicting phylogenetic signals associated with genetic recombination. The statistical confidence of these structures was evaluated through bootstrap resampling (10,000 replicates), with only those exhibiting both bootstrap and fit scores of ≥80 retained as significant evidence of recombination. To ensure further that these signals reflected true recombination rather than convergent evolution, we subjected the recombinant *msrA* alleles to the Pairwise Homoplasy Index (PHI) test (Bruen et al., 2006). This test served as an additional critical control, enabling us to distinguish genuine recombination events from homoplasious patterns that could arise through convergent mutations across taxa.

### 2.5 MOB-suite–based characterization and mobility modeling of *msrA*-carrying plasmids

To characterize *msrA*-carrying plasmids, their DNA sequences were analyzed using the MOB-suite module *mob_typer* (Robertson and Nash, 2018). This analysis inferred key plasmid features, including size, GC content (%), predicted mobility (non-mobilizable, mobilizable, or conjugative), and primary mobility cluster. The association between *msrA* allele type and plasmid mobility class was assessed using a chi-square test of independence, with effect size quantified by Cramér’s *V* (Cramér, 1946). Standardized residuals were calculated to identify specific allele–mobility combinations contributing to the association (Agresti, 2013). To mitigate bias due to sparse categories, a secondary analysis was performed by grouping low-frequency alleles.

To evaluate whether allele identity independently predicts plasmid mobility, logistic regression models were fitted. Due to the low frequency of conjugative plasmids, mobility was modeled as a binary outcome (mobilizable vs. non-mobilizable). Predictor variables included allele identity (categorical), plasmid size (continuous), and plasmid GC content (continuous), with continuous variables standardized prior to analysis. Model comparisons were performed using likelihood-ratio tests, and effect sizes were reported as odds ratios (OR) with 95% confidence intervals (Hosmer et al., 2013). Differences in plasmid size between mobility groups were assessed using non-parametric tests. The Mann–Whitney U test was applied for binary comparisons, and the Kruskal–Wallis test was used for comparisons across all three mobility classes, followed by pairwise comparisons where appropriate (Conover, 1999).

Allele host breadth was defined as the number of distinct species and genera in which each allele was detected. Alleles were classified as species-shared or genus-shared based on their presence across taxa. To assess the relationship between allele dissemination and plasmid background diversity, the number of distinct plasmid primary clusters associated with each allele was calculated. Associations between host breadth and plasmid cluster diversity were evaluated using Spearman’s rank correlation (Spearman, 1904), and differences between species-shared and species-restricted alleles were assessed using the Mann–Whitney U test. Evidence for plasmid-mediated dissemination of *msrA* was inferred from the occurrence of identical allele–plasmid-cluster combinations across multiple host species or genera. All statistical analyses were performed using standard approaches for categorical and non-parametric data. All tests were two-sided, and *p*-values < 0.05 were considered statistically significant.

## 3 Results

### 3.1 Polymorphisms and signatures of selection and conservation across *msrA*

**Figure 1:**
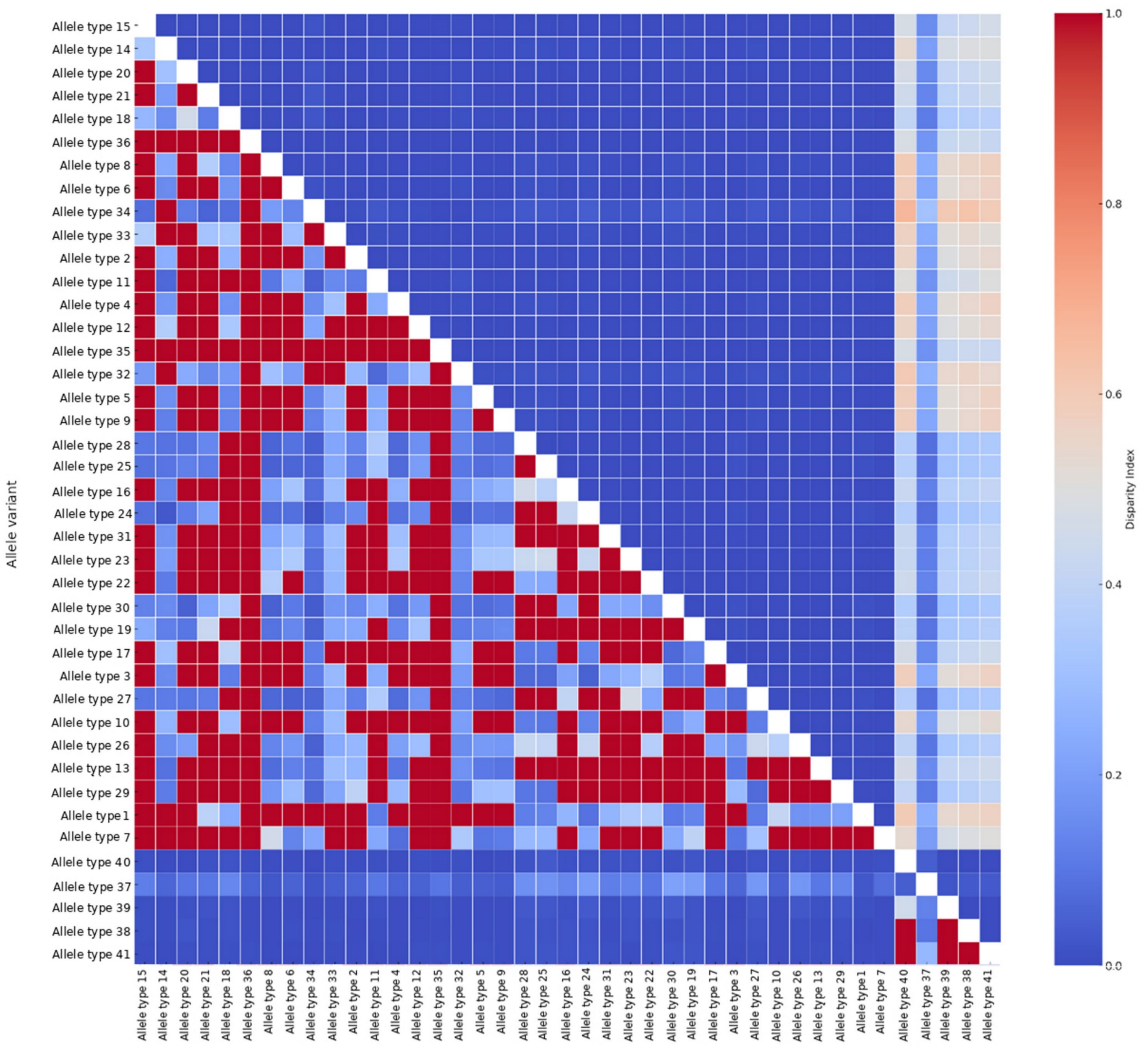
The Pairwise Disparity Index heatmap of substitution pattern homogeneity among *msrA* allelic variants.

The DNA sequence variation analysis classified 601 CDSs of the *msrA* gene into 41 distinct allelic types (Supplementary Table S1). Among 1,467 nucleotide sites analyzed for the *msrA* gene, 1,151 were invariable, 233 were parsimony-informative, and 83 were singletons. The estimated values, for allelic diversity, nucleotide diversity (*π*), and the average number of pairwise nucleotide differences (*k*) across this gene, were 1.000 ± 0.005, 0.03878 ± 0.01045, and 56.884, respectively. The subsequent correction of π using the Jukes-Cantor model yielded an adjusted estimate of 0.04218. In addition, according to the Disparity Index (DI) Test-generated values, a majority of the *msrA* allelic pairs exhibited low to moderate heterogeneity in nucleotide substitution patterns. However, certain alleles (e.g., the *msrA* allelic types 37–41, all exclusive to *Staphylococcus arlettae*) demonstrated markedly higher divergence (DI > 0.5). Figure 1 presents a heatmap of pairwise DI values illustrating the substitution pattern heterogeneity among the *msrA* alleles. In addition, we estimated the patterns of nucleotide substitutions in the alleles of *msrA* collectively. As shown in Table 1, the transition rates exceeded the transversion rates, with the highest ML- MCL-derived probabilities observed for G → A (20.72 and 19.52) and C → T (20.61 and 21.7) transitions in this gene, respectively. The overall transition/transversion bias (*R*) was 1.36, with base-specific rate ratios of *k₁* = 2.47 (for purines) and *k₂* = 3.97 (for pyrimidines). In the DnaSP analysis, Watterson’s *θ*, estimated from segregating sites, was 0.05035 per site, while θ calculated from the total number of mutations (Eta) was slightly higher at 0.05592 per site, indicating comparable levels of nucleotide diversity inferred from both estimators. The close agreement between these estimators is in concordance with sequence variation that is not strongly influenced by extreme allele-frequency distortions or pronounced localized mutational hotspots.

**Table 1:** The ML and MCL estimates of transition and transversion substitution probabilities for the *msrA* gene.

Substitutions	**ML/MCL probability (*r*) of substitution**			
	**A**	**T/U**	**C**	**G**
A	–	*5.47/5.47*	*2.91/2.92*	**8.58/8.09**
T/U	*7.90/7.90*	–	**10.98/11.56**	*3.27/3.27*
C	*7.90/7.90*	**20.61/21.7**	–	*3.27/3.27*
G	**20.72/19.52**	*5.47/5.47*	*2.91/2.92*	–

Rates of different transitional substitutions are shown in bold and those of transversional substitutions are shown in italics.

**Figure 2:**
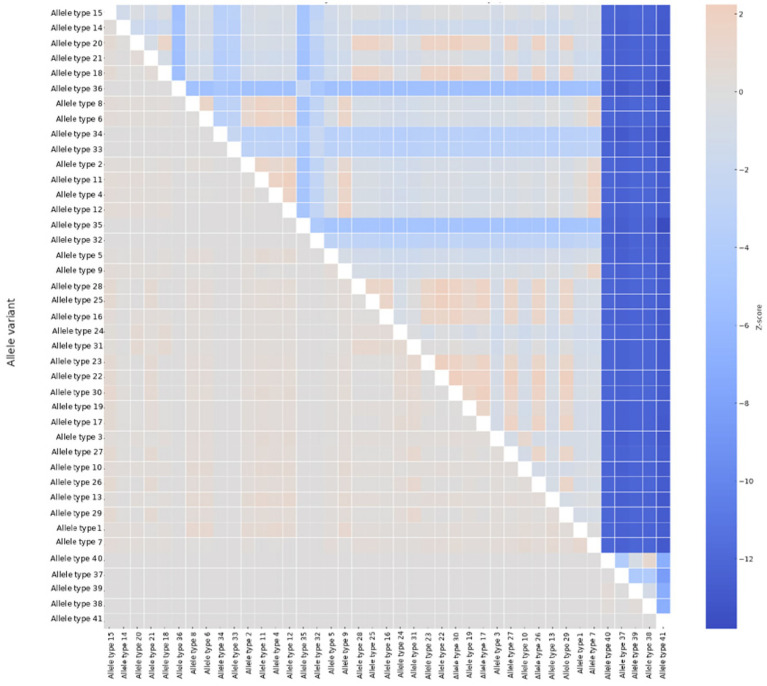
The heatmap of pairwise codon-based *Z*-test scores for neutrality across the *msrA* gene allelic types.

Using MEGA12 and DnaSP, we also performed neutrality and selection analyses of the targeted *msrA* gene. The summarized data obtained from the DnaSP analysis are presented in Table 2. As shown, a marked discrepancy between π_synonymous (0.11284) and π_nonsynonymous (0.01990) yielded a π_a_/π_s_ ratio of ~0.169, consistent with strong purifying selection across this gene; The Tajima’s D values were consistently negative but not statistically significant (e.g., *D* = −1.14212, *p* > 0.10), while Fu’s Fs was strongly negative (−11.856, *p* = 0.0001). In addition, the Jukes-Cantor-corrected *d_N_-ds* was −0.138170 for *msrA* when analyzed using the Nei–Gojobori method implemented in MEGA12, indicating pervasive purifying selection. The *Z*-scores obtained from the codon-based *Z*-test for neutrality revealed a widespread pattern of negative values across most pairwise comparisons (Figure 2), supporting pervasive purifying (negative) selection. In contrast, the Fisher’s Exact test results (*p*-values ranged from 0.3 to 1.0) did not demonstrate statistically significant deviations from neutrality. This outcome is more plausibly attributable to the conservative behavior and reduced power of the above test in pairwise comparisons, rather than representing a genuine contradiction to the strong evidence for purifying selection acting on this gene. However, using MEME, we identified four codons (positions 93, 100, 163, and 177) exhibiting signatures of episodic positive selection (*p* ≤ 0.0396), affecting a very limited number of *msrA* allelic types (Table 3). Furthermore, under synonymous rate variation, the aBSREL model identified a single branch corresponding to allele 1, providing additional evidence for episodic diversifying selection (LRT = 13.04, *p* = 0.028). A small proportion of sites (~0.45%) were inferred to be under strong positive selection (*ω* ≈ 814).

**Table 2:** DNA polymorphism, nucleotide diversity, and neutrality test statistics for the *msrA* gene.

**Parameter**	**Estimate**	***P*-value**
π synonymous (π_s)	0.11284	–
π nonsynonymous (*π*_a)	0.019904	–
π_a/ π_s ratio	≈ 0.169	–
Tajima’s *D* (overall)	−1.14212	>0.10
Tajima’s *D* (coding region)	−1.16170	>0.10
Tajima’s *D* (synonymous)	−1.01459	>0.10
Tajima’s *D* (non-synonymous)	−1.28702	>0.10
Tajima’s *D* (silent)	−1.01459	>0.10
Tajima’s *D* (segregating sites)	−0.85524	>0.10
Fu’s *F*s	−11.856	0.0001
Fu and Li′s *D*	−0.46175 to –0.60493	>0.10
Fu and Li′s *F*	−0.73989 to –0.95477	>0.10

**Table 3:** The MEME-determined codon sites exhibiting episodic positive selection across the *msrA* Gene.

**Codon position**	LRT*	**Branches #**	**Common substitutions**	***P*-value**
93	3.101	1	[1]Ctc > Atc,ctC > ctT	0.0396
100	4.108	2	[1]acC > acT,aCc > aTc, Acc > Gcc	0.0297
163	6.275	1	[1]gaC > gaT, GAc > TCc	0.0099
177	3.908	1	[1]aCa > aAa	0.0099

LRT*-Likelihood Ratio Test-derived value.

**Figure 3:**
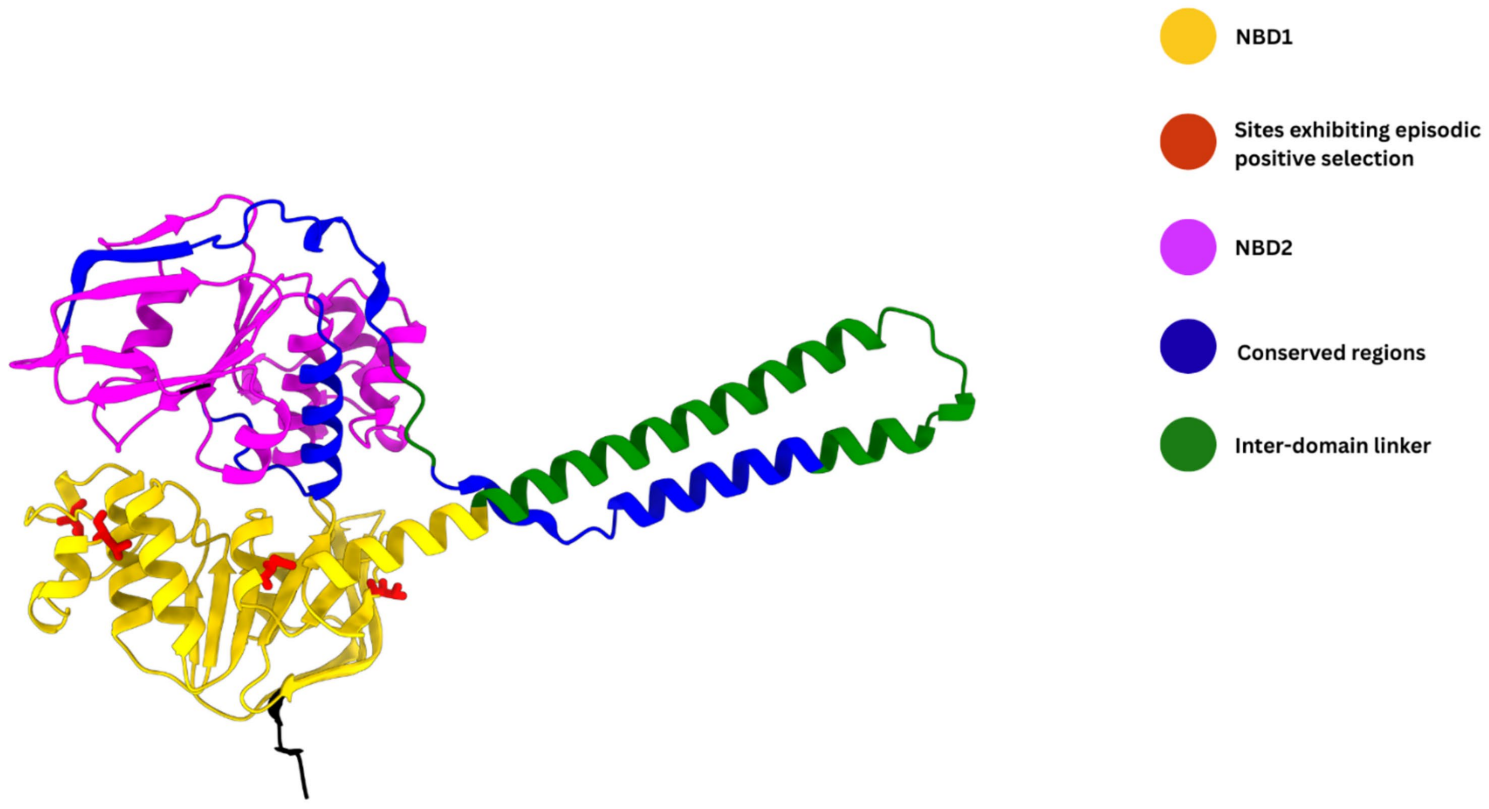
The overlapping relationships between the DnaSP-determined highly conserved regions and the functional domains in the Staphylococcal *msrA* protein.

The FoldX analysis of the MEME-inferred sites under positive diversifying selection revealed that at position 93, the conservative L93I substitution was predicted to be strongly destabilizing (ΔΔG = +3.95 kcal/mol), indicating sensitivity to subtle changes in side-chain geometry and local packing; at position 100, substitutions from threonine to isoleucine or alanine resulted in context-dependent stability effects (ΔΔG = −0.38 and +1.15 kcal/mol, respectively), despite the both amino acid changes involving a shift toward increased hydrophobicity and reduced polarity. In contrast, the D163S substitution led to a pronounced destabilizing effect (ΔΔG = +2.14 kcal/mol), consistent with the loss of negative charge and potential disruption of electrostatic interactions. Finally, the T177K substitution had a negligible impact on stability (ΔΔG = −0.16 kcal/mol). The sites identified as being under positive diversifying selection were mapped and subsequently visualized on both the MsrA protein (Figure 3) the respective ML tree branches (Supplementary Figure S1), elucidating genetic relationships among the *msrA* allelic types.

In the DNA sequence analysis of 41 allelic types, we identified three significantly conserved regions with the size ranging from 65 bps to 76 bps in length (Table 4). These regions exhibited high conservation values (0.884–0.908) and very high site-averaged homozygosity (*H* = 0.975–0.980), indicating strong sequence uniformity across the alleles. In the subsequent analysis, we mapped these regions into the functional domains identified by UniProt to determine if there was any overlapping relationship between these regions and NBD1, NBD1, and/or their inter-domain linker across the amino acid (AA) sequences of the MsrA protein (UniProt accession: P23212). No overlap was observed between any of the conserved regions and NBD1 (AA positions: 6th–199th) in the gene. Conserved region 1 (AA positions: 254th–279th) was entirely confined to the inter-domain linker (AA positions: 200th–298th), covering 26.26% of this domain in MsrA. Conserved region 2 (AA positions: 284th–307th) partially overlapped (coverage: 15.15%; AA positions: 284th–298th) the inter-domain linker, extending further (with 4.76% of coverage) to NBD2 (AA positions: 299th–487th). Conserved region 3 (AA positions: 435th–456th) was found to be fully contained within NBD2 in this gene. Figure 3. Provides a graphical representation of the overlapping relationships between the above conserved regions 1–3 and the functional domains determined by UniProt within the MsrA protein.

**Table 4:** The significantly conserved regions determined by DnaSP across the *msrA* gene.

Region	Positions	Length (bp)	Conservation	Site-averaged homozygosity (H)	*P*-value
1	760–835	76	0.908	0.980	0.0032
2	851–919	69	0.884	0.975	0.0228
3	1,303–1,367	65	0.892	0.978	0.0172

### 3.2 Allelic distribution of *msrA* in *Staphylococcus*

Among the 41 identified *msrA* allelic types, five—allele 19 (*n* = 415), allele 16 (*n* = 57), allele 2 (*n* = 41), allele 24 (*n* = 16), and allele 17 (*n* = 12)—were predominant, collectively accounting for 90.02% of all CDSs analyzed (Supplementary Table S1). The alleles designated as predominant differed markedly in their distribution among carrier species. Allele 19 was carried almost exclusively by *S. aureus* (412/415; 99.28%), with only single occurrences in *Staphylococcus haemolyticus* and *Staphylococcus hominis* (1/415; 0.24% each). Similarly, allele 24 was found exclusively in *S. aureus* strains (16/16; 100%). Allele 17 was also predominantly associated with *S. aureus* (10/12; 83.33%), with limited representation from *S. haemolyticus* and *Staphylococcus epidermidis* (each 8.33%), exhibiting a relatively narrow host range. In contrast, allele 16 showed a substantially broader intra-genus distribution within *Staphylococcus*, occurring in *S. aureus* (19/57; 33.33%), *S. haemolyticus* (29.82%), *S. epidermidis* (29.82%), *S. hominis* (3.51%), *Staphylococcus saprophyticus* (1.75%), and *Staphylococcus capitis* (1.75%). Allele 2 displayed the broadest taxonomic host range, spanning multiple *Staphylococcus* species and extending beyond the genus. It was frequently detected in *S. epidermidis* (17/41; 41.46%), followed by *S. aureus* (21.95%), *Staphylococcus warneri* (9.76%), and *S. hominis* (7.32%), with single occurrences in *S. haemolyticus*, *Staphylococcus argenteus*, *Staphylococcus caprae*, and *Staphylococcus massiliensis*. Additionally, allele 2 was detected in three non-*Staphylococcus* organisms, each represented by a single strain from yet-unidentified species of *Streptococcus*, *Enterococcus,* and *Corynebacterium*. Notably, allele 7 was shared by *S. warneri* (*n* = 1) and *Citrobacter portucalensis* (*n* = 1), with allele 11 represented singly by a strain from the unidentified species of *Pseudomonas*. As shown in the ML tree, depicting genetic relationships among the *msrA* allelic types (Supplementary Figure S1), this allele clustered most closely with allele 1 carried exclusively by *S. epidermidis*.

We next examined the genomic localization of the predominant *msrA* alleles across the carrier organisms analyzed. The vast majority of *msrA* CDSs harboring allele 19 (333/415; 80.24%) were plasmid-associated, with a much smaller fraction located on chromosomes (19.28%). No significant difference was revealed in the frequency of distribution of allele 16 between plasmids (30/57; 52.63%) and chromosomes (27/57; 47.37%). Allele 2 was predominantly plasmid-borne (31/41; 75.61%), with relatively few chromosomal instances (14.63%). In contrast, allele 24 was exclusively chromosomal (16/16; 100%), whereas allele 17 was primarily associated with plasmids (9/12; 75.00%), with the remaining instances located on chromosomes (25.00%). Overall, 25 allelic types were detected exclusively on plasmids (alleles 4–10, 12–15, 18, 20–23, 25–30, and 32–34), while 10 allelic types (alleles 1, 24, and 31–41) were found only on chromosomes. The remaining five allelic types (alleles 2, 3, 16, 17, and 19) were observed in both plasmid and chromosomal contexts. Allelic overlap among conspecific isolates within individual *Staphylococcus* species is expected under transmission of *msrA* via clonal expansion and intra-species recombination. In contrast, allelic overlap observed between different species or genera provides strong evidence for interspecies and intergeneric recombination, indicating additional routes of *msrA* dissemination both within and beyond the *Staphylococcus* genus.

### 3.3 Plasmid-induced dissemination of *msrA*

The results of the *msrA*-carrying plasmids classification, including, but not limited to, plasmid-predicted mobility and primary clusters inferred by the MOB-suite module *mob_typer*, are detailed in Supplementary Table S1. The chi-square test of independence revealed a highly significant association between the *msrA* allele type(s) and plasmid mobility (χ^2^ = 728.52, df = 87, *p* < 0.001). The effect size was large (Cramér’s *V* = 0.73), indicating strong structuring of allele distribution across mobility categories. The standardized residual analysis identified the specific allele–mobility combinations driving this association. Specifically, allele 10 (residual = +18.23) and allele 8 (+10.52) were strongly enriched in conjugative plasmids, indicating preferential association with highly mobile genetic elements. In contrast, several alleles, including alleles 17 (+6.36), 16 (+5.16), 2 (+5.01), and 14 (+4.81), were significantly enriched in non-mobilizable plasmids, exhibiting preferential association with plasmids lacking autonomous transfer capability. To account for potential bias introduced by the low-frequency alleles, the secondary analysis was performed with the rare alleles grouped. This yielded a more conservative but still significant association (χ^2^ = 193.88, df = 6, *p* < 0.001; Cramér’s *V* = 0.46), confirming that the observed relationship is robust, although partially influenced by sparse categories.

To assess whether allele identity remained associated with plasmid mobility independently of plasmid characteristics, the logistic regression models were fitted including plasmid size and GC content (%) as covariates. Due to the low number of conjugative plasmids (*n* = 4), inference was based on a binary comparison between mobilizable and non-mobilizable plasmids. The inclusion of the allelic profiles significantly improved model fit compared to a model including only plasmid size and GC content (likelihood-ratio χ^2^ = 143.68, df = 3, *p* < 0.001), demonstrating that allele identity is an independent predictor of plasmid mobility. The plasmids carrying allele 19 were strongly associated with the mobilizable plasmids relative to the reference group, whereas the other allele groups did not show significant differences. Plasmid size also contributed independently to mobility classification. The inclusion of plasmid size significantly improved model fit (likelihood-ratio χ^2^ = 24.48, *p* < 0.001), with larger plasmids showing higher odds of being classified as transmissible (OR = 2.07 per standard deviation increase, 95% CI: 1.45–2.97, *p* < 0.001). In contrast, GC content did not show a significant independent association after adjustment. In the unadjusted analyses, plasmid size did not differ significantly between the transmissible and non-transmissible plasmids (Mann–Whitney *U* test, *p* = 0.511), with similar median sizes observed (26,410 bp vs. 27,066 bp, respectively). When considering all three mobility classes, a significant overall difference in plasmid size was observed (Kruskal–Wallis test, *p* = 0.0028). However, this effect was primarily driven by a small number of large conjugative plasmids (*n* = 4; median 61,522 bp), while no significant difference was observed between mobilizable and non-mobilizable plasmids (*p* = 0.588). These findings indicate that plasmid size is not a strong standalone discriminator of mobility class.

Furthermore, to investigate whether the plasmids had contributed to the interspecies and intergeneric distribution of the *msrA* alleles, we integrated allele host range, plasmid mobility, and the plasmid primary-cluster assignments derived from MOB-suite analysis. Among 41 *msrA* alleles identified, 8 (19.5%) were detected in more than one species, whereas only 2 alleles (4.9%) were observed across more than one genus. All the alleles shared across the species were associated with plasmids in at least one instance, and the majority (7/8) were observed on the mobilizable or conjugative plasmids. A strong positive correlation was observed between allele host breadth and plasmid background diversity, as measured by the number of the distinct plasmid primary clusters carrying each allele (Spearman *ρ* = 0.85, *p* = 2.97 × 10^−9^). Consistently, the alleles, detected across multiple species, were associated with significantly more plasmid clusters than the species-restricted alleles (median 3 vs. 1; Mann–Whitney *p* = 6.91 × 10^−6^). In this light, no significant association was observed between host breadth and plasmid size, or GC content. Several allele–plasmid-cluster combinations were shared across multiple *Staphylococcus* species, illuminating most likely plasmid-mediated interspecies dissemination of *msrA*. For example, allele 2 was detected on the cluster AD281 in *S. epidermidis*, *S. hominis*, and *Staphylococcus warneri*, as well as on the cluster AA072 observed in *S. aureus*, *S. epidermidis*, *S. haemolyticus*, and *S. hominis*. Similarly, allele 17 on cluster AD279 was observed in *S. capitis*, *S. epidermidis*, and *S. hominis*, while allele 16, associated with the same cluster, was found in *S. epidermidis*, *S. haemolyticus*, and *S. saprophyticus*. The additional shared combinations included allele 19 on the cluster AA840 in *S. aureus* and *S. hominis*, and allele 16 on the clusters AB125 and AD280 spanning *S. epidermidis*, *S. hominis*, and *S. haemolyticus*. In contrast, evidence for intergeneric dissemination was limited. Only two alleles (alleles 2 and 7) were detected across the different genera, including occurrences in *Enterococcus*, *Streptococcus*, and *Corynebacterium* species, many of which lacked confirmed plasmid origin. Furthermore, only a single plasmid cluster (AD280) spanned more than one genus, including *C. portucalensis* and *Staphylococcus* species, and was classified as non-mobilizable.

### 3.4 LD landscape and recombination patterns in *msrA*

**Figure 4:**
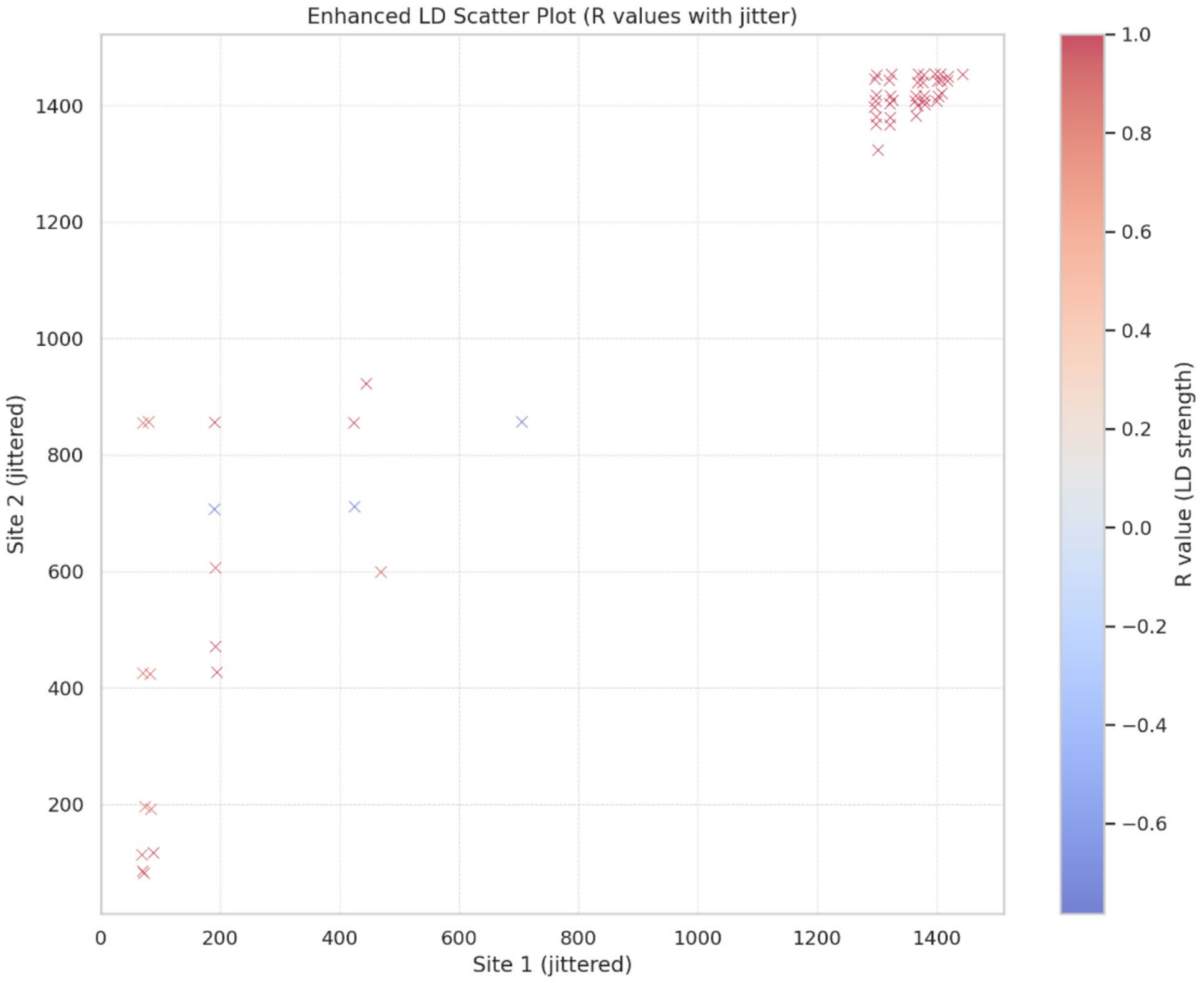
The scatter plot of pairwise LD (R) patterns among the polymorphic sites determined across the DNA sequence alignment of the *msrA* gene allelic variants.

The LD patterns were assessed across a 1,467-bp DNA sequences alignment of 41 allelic types of the *msrA* gene, encompassing 281 polymorphic sites, and yielding 39,340 pairwise comparisons (Supplementary Table S2). The normalized LD coefficient (D′) frequently reached absolute values of 1.000, indicating complete (or complete inverse) LD between many site pairs, including pairs separated by more than 1,000 bp. Notably, several comparisons produced the maximum possible χ^2^ value of 41.000, consistent with perfect association given the observed sample size and allele frequencies. Site 7 exhibited particularly strong LD with multiple distant positions, including sites 42, 43, 60, and 66 (separated by 35–59 bp), as well as sites 984 (977 bp), 1,125 (1,118 bp), 1,223 (1,216 bp), 1,332 (1,325 bp), 1,374 (1,367 bp), and 1,377 (1,370 bp). In these comparisons, D′ values were equal to or very close to 1.000, accompanied by highly significant Fisher exact test results (*p* ≤ 0.001 in most cases) and χ^2^ values frequently reaching or approaching 41.000. A comparable pattern of extensive complete or near-complete LD was observed in the 3′ region of the DNA alignment the *msrA* gene alleles (positions ~1,365–1,459). The pairwise comparisons within this block yielded *D*′ = 1.000, χ^2^ = 41.000 (or similarly high values), and Fisher exact test *p*-values approaching zero, with strong associations persisting across distances of up to ~94 bp. Overall, LD showed little evidence of decay with increasing physical distance across substantial portions of the *msrA* alignment. Figure 4 displays a scatter plot of pairwise LD (*R*) values among polymorphic sites across the *msrA* allelic variants, restricted to comparisons supported by both Fisher exact test and χ^2^ statistics; here, we show the identified dense cluster of site pairs exhibiting high LD values (*R* > 0.8) predominantly located in the upper-right quadrant of the scatter plot. The prevalence of long-range complete LD involving key polymorphic sites, together with the large number of highly significant pairwise comparisons (hundreds with *p* < 0.001), is consistent with limited effective recombination, and the dominance of one or a very small number of haplotype backgrounds within the dataset.

**Figure 5:**
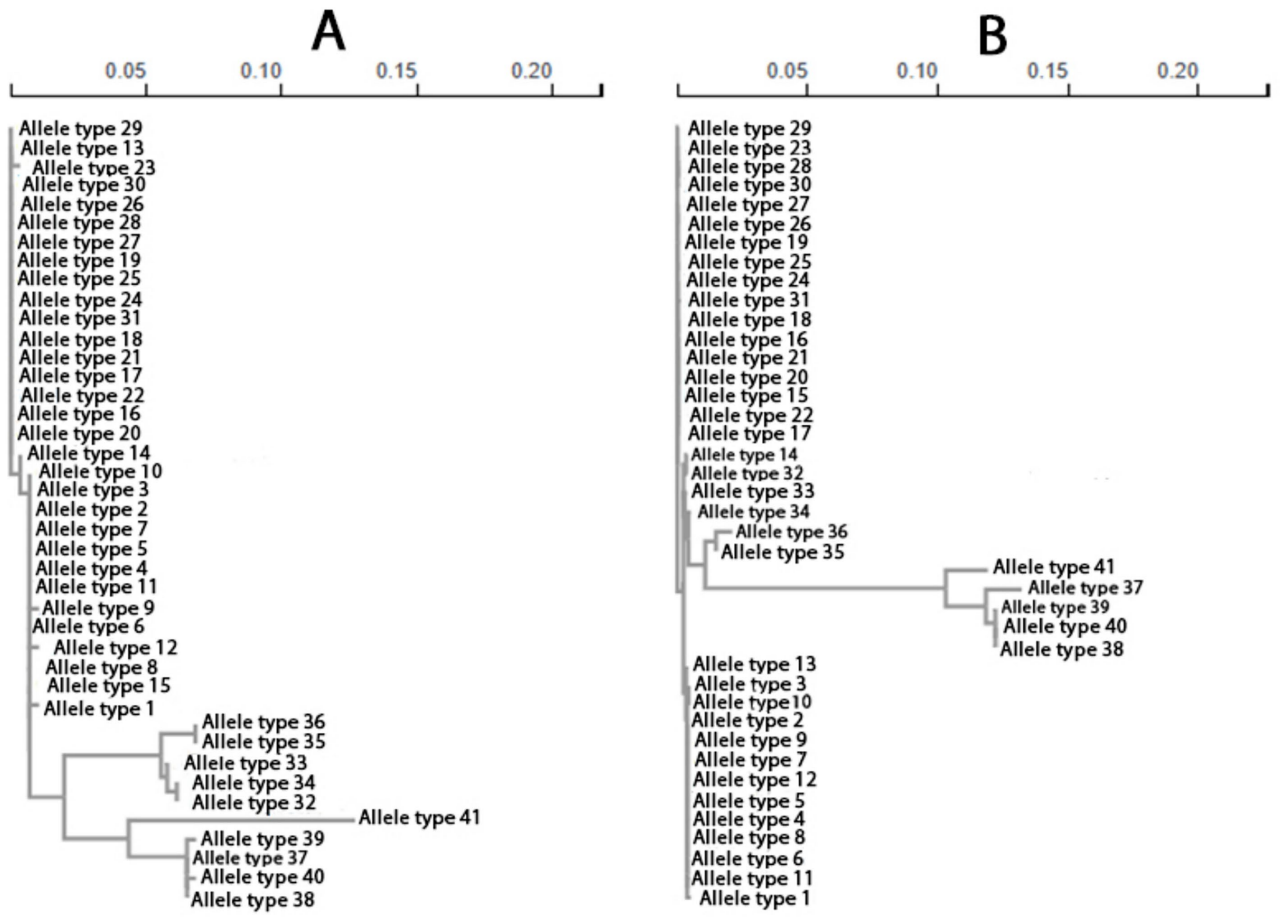
Incongruent ML phylogenies inferred from GARD-determined recombinant regions of the *msrA* gene. **(A)** ML phylogeny inferred from the GARD-determined recombinant region spanning nucleotide positions 1–170 of *msrA*. **(B)** ML phylogeny inferred from the GARD-determined recombinant region spanning nucleotide positions 171–1,467 of *msrA.*

In addition, as shown in Table 5, the average squared correlation coefficient ZnS was 0.3600, indicating moderate to moderately high LD on average. The normalized LD measure Za reached 0.4204, while ZZ was only 0.0604, further supporting the observed patterns of minimal decay of LD with physical distance and the persistence of long-range associations. Wall’s B (0.2464) and Wall’s *Q* (0.2811) further indicated that a substantial proportion of informative site pairs were fully compatible, consistent again with limited historical recombination and the presence of one or a small number of predominant haplotype backgrounds. These inferences of genetic recombination were further strongly supported by the ML–based phylogenetic incongruences among the specific allelic types (Figure 5), together with the recombination breakpoints (coordinate ranges: 1–170 and 171–1,467) identified across *msrA*, using GARD.

**Table 5:** Summary of linkage disequilibrium statistics for the *msrA* gene.

**Statistic**	**Estimate**
ZnS	0.3600
Za	0.4204
ZZ	0.00604
Wall’s *B*	0.2464
Wall’s *Q*	0.2811

### 3.5 RDP4-infered interspecies and intergeneric recombination events of *msrA*

We applied the array of recombination detection algorithms implemented in RDP4 to identify putative donors and recipients of *msrA*, and to elucidate the transmission trajectories of this gene within *Staphylococcus* and beyond. Table 6 summarizes the RDP4 results, including the recombination beginning and end breakpoints detected within *msrA*, and the representative strains of the inferred donor and recipient species for the recombined loci. The more detailed recombination inferences, determined by RDP4, are provided in Supplementary Table S3.

**Table 6:** The networks of representative donors and recipients of the *msrA* genetic loci determined by RDP4.

Recombination breakpoint positions across *msrA* of recombinant organism(s)		Representative recombinant organism(s) (GenBank ID)	Representative major donor(s)	Representative minor donor(s)	*P* value generated by algorithm used for detecting LGT event(s)					
Beginning	End	Recombinant clusters	Major donor clusters	Minor donor clusters	GENCONV	Bootscan	MaxChi	Chimaera	SiScan	3Seq
39	1,054	Î1	Í1	Ï1	0.011	0.001	7.10E-06	7.14E-06	3.66E-07	6.49E-06
319	1,198	Î2	Í2	Ï2	NS*	NS*	0.004	0.030	0.005	8.43E-05

Î1 – Species representative recombinant strain followed by its GenBank accession ID, and No. of (*n*) of other respective recombinant-conspecific(s): *S. aureus* MsrSA (AB013298.1) (*n* = 3); *Staphylococcus chromogenes* 1,401 (CP046029.1) (*n* = 1); *Staphylococcus saprophyticus* Dog122 (CP120075.1) (*n* = 1). Í1 – Species representative major donor strain followed by its GenBank accession ID, and No. of (*n*) of other major donor-conspecifics: *S. epidermidis* SP1 (KR230047.1) (*n* = 1); *Staphylococcus equorum* FDAARGOS_1149 (CP068070.1.1) (*n* = 4); *Staphylococcus xylosus* 14BME10 (CP154367.1) (*n* = 2); *S. aureus* 0831 (CP104021.1) (*n* = 456); *Staphylococcus haemolyticus* 114126 (OZ330104.1) (*n* = 17); *Staphylococcus hominis* C5 (CP093540.1) (*n* = 2); *Staphylococcus capitis* BN2 (CP042341.1) (*n* = 1); *Staphylococcus cohnii* Dog166 (CP119999.1) (*n* = 1); *Staphylococcus warneri* SWO (CP033100.1) (*n* = 1); *Citrobacter portucalensis* F2221 (CP137483.1) (*n* = 1). Ï1–Species representative minor donor strain followed by its GenBank accession ID, and No. of (n) of other minor donor-conspecifics: *Staphylococcus cohnii* WT1082 (CP188078.1) (*n* = 2); Î2 - Species representative recombinant strain followed by its GenBank accession ID, and No. of (*n*) of other respective recombinant-conspecific(s): *Staphylococcus saprophyticus* GDY8P168P (CP065799.1) (*n* = 1). Í2–Species representative major donor strain followed by its GenBank accession ID, and No. of (*n*) of other major donor-conspecifics: *Staphylococcus cohnii* SDAQ-1 (CP126540.1) (*n* = 1). Ï2–Species representative minor donor strain followed by its GenBank accession ID, and No. of (*n*) of other minor donor-conspecifics: *Staphylococcus equorum* ADH2-898 (CP085227.1) (*n* = 4); *Staphylococcus xylosus* 14BME10 (CP154367.1) (*n* = 2); S. aureus UNC_SA54 (CP132366.1) (*n* = 4); *Staphylococcus capitis* Sc1365665 (CP145252.1); (*n* = 1); *Staphylococcus epidermidis* HD75-1 (CP052944.1) (*n* = 1); *Staphylococcus haemolyticus* NY5 (CP078159.1) (*n* = 1); *Citrobacter portucalensis* F2221 (CP137483.1) (*n* = 1). NS*–Not significant.

Two distinct recombination breakpoint clusters were identified within the *msrA* gene, spanning nucleotide positions (ntps) 39–1,054 and 319–1,198. As shown in Table 6, the largest number of putative major donor strains (*n* = 456) belonged to *S. aureus* from the *Staphylococcus* species network involved in interspecies recombination events of *msrA*. These events were responsible for the transmission of one *msrA* internal locus (ntps: 39–1,054) to *S. chromogenes* and *S. saprophyticus*. In addition, several other species within this network—including, but not limited to, *S. epidermidis*, *S. equorum*, *S. haemolyticus*, *S. hominis*, and *S. cohnii*—were inferred to be the putative major donors of this locus to *S. aureus*, *S. chromogenes*, and *S. saprophyticus*. Notably, *C. portucalensis* was also inferred as a major donor for these three species, indicating intergeneric recombination events involving *msrA*.

Both interspecies and intergeneric recombination events were likewise detected for the second *msrA* locus (ntps: 319–1,198), with the majority of these species acting as donors to *S. saprophyticus*. It should be noted that the inferred recombination breakpoints associated with these events do not necessarily imply genetic exchange limited to internal *msrA* loci; in some cases, they may reflect transmission of the entire gene. The *p*-values generated by RDP4 across most recombination detection algorithms supported the robustness of these inferences.

**Figure 6:**
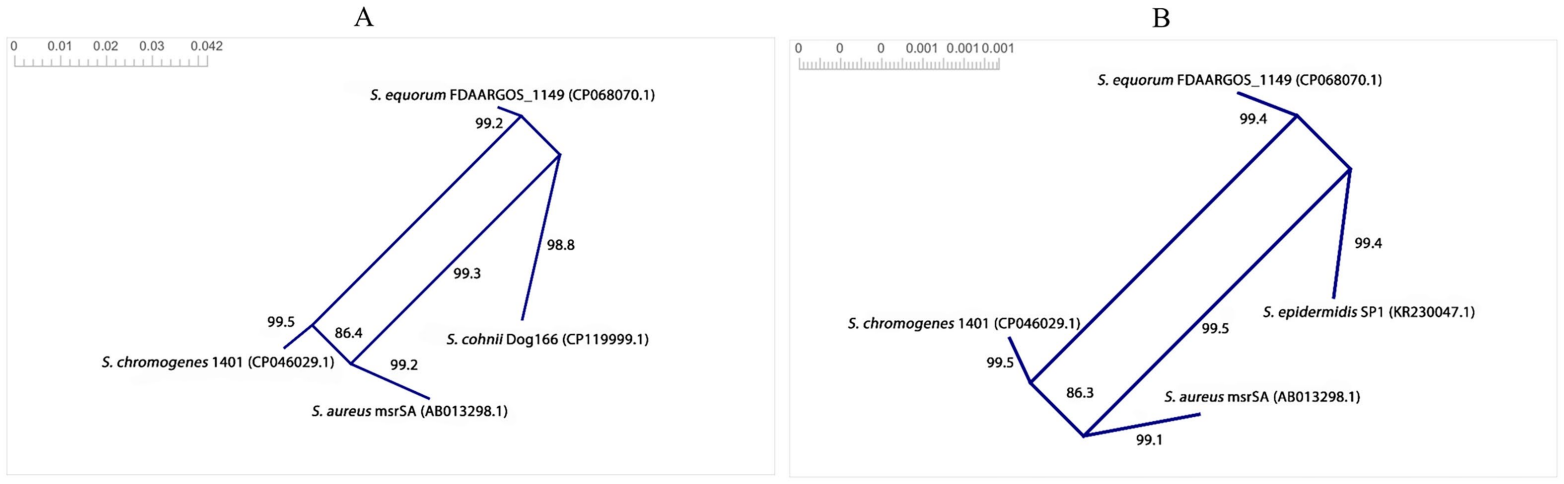
The SplitsTree-generated parallelograms exhibiting the interspecies recombination events of *msrA* in *Staphylococcus*: **(A)** The strain representatives of *S. aureus*, *S. equorum*, *S. chromogenes*, and *S. cohnii*, exchanging *msrA*; **(B)** The strain representatives of *S. aureus*, *S. equorum*, *S. chromogenes*, and *S. epidermidis*, exchanging *msrA*.

Although the split decomposition method, implemented in SplitsTree (v6.7.0), cannot identify donors and recipients, we additionally applied it to the same subset of DNA sequences to obtain complementary statistical evidence for interspecies and intergeneric recombination events of *msrA*. Because parallel nucleotide substitutions inferred by split decomposition may reflect conflicting evolutionary histories arising from convergent evolution that mimics genetic recombination, we reexamined the putatively recombined *msrA* loci using the PHI test to detect homoplasy patterns. Figures 6A,B shows the SplitsTree-generated parallelograms that were exclusively and strongly supported by both very high bootstrap values (with the highest fit reaching 100) and robust probability estimates from the PHI test. In contrast to other parallelograms that lacked PHI test support (data not shown), these structures provide additional evidence for genetic exchange of *msrA* among *S. aureus*, *S. epidermidis*, *S. equorum*, *S. chromogenes*, and *S. cohnii*, representing part of the donor–recipient network underlying this gene transmission within *Staphylococcus*.

## 4 Discussion

In this study, we provide a comprehensive analysis of the evolutionary landscape of *msrA* in *Staphylococcus*, using a large and taxonomically diverse dataset comprising of 601 CDSs condensed into 41 allelic types. We reconstructed the evolutionary dynamics of *msrA* and modeled the donor–recipient networks underlying its dissemination across *Staphylococcus* species, with particular emphasis on the role of interspecies and intergeneric recombination events. Accordingly, the following discussion is framed around two key dimensions: the evolutionary dynamics of *Staphylococcus msrA* and the donor–recipient interactions that facilitate its transmission within and beyond this genus.

### 4.1 Evolutionary dynamics of *msrA*

The *msrA* gene exhibits a relatively low overall nucleotide diversity (*π* ≈ 0.0388–0.0422, *k* ≈ 56.9) across the diverse bacterial hosts, with five predominant alleles accounting for the vast majority of occurrences of this gene within *Staphylococcus*. This pattern suggests strong selective constraints limiting diversification, consistent with the pervasive purifying (negative) selection inferred from the multiple lines of evidence: a low π_a_/π_s_ ratio (~0.169), negative (though non-significant) Tajima’s D values, strongly negative Fu’s Fs (−11.856, *p* = 0.0001), and negative *d_N_-d_S_* (−0.138170). These metrics collectively indicate that purifying selection has eliminated most deleterious nonsynonymous changes, preserving core functionality of the MsrA protein as an ABC-F subfamily ribosomal protection determinant that shields the peptidyl transferase center from macrolide and streptogramin B antibiotics. Furthermore, the above evolutionary patterns—particularly the strongly negative Fu’s Fs and the skewed allele-frequency distribution—may also reflect demographic expansion, genetic hitchhiking, or selective sweep dynamics associated with the rapid spread of successful *msrA* allelic backgrounds. The observed transition/transversion bias in *msrA* (*R* = 1.36), with elevated purine- and pyrimidine-specific ratios, aligns with mutational patterns typical of genes under functional constraint, as commonly seen in housekeeping and essential bacterial genes (Lind and Andersson, 2008). This holds despite the role of *msrA* as an acquired accessory antimicrobial resistance determinant, mirroring the patterns observed in the *tetM* gene involved in tetracycline-resistance, where strong transition bias co-occurs with pervasive purifying selection (Tsiklauri et al., 2025; Kobakhidze et al., 2026).

The structural analysis of the MEME-inferred sites suggests that positively selected substitutions can have heterogeneous effects on protein stability, ranging from strongly destabilizing (e.g., L93I and D163S) to nearly neutral (e.g., T177K). In this light, it is noteworthy that, even conservative or physicochemically subtle changes can lead to substantial destabilization, underscoring the sensitivity of local structural environments of proteins. These observations support the view that positive (diversifying) selection does not necessarily favor increased protein stability, but rather reflects optimization of overall organismal fitness. In this context, functional advantages, such as altered interactions or increased adaptive flexibility, may outweigh structural costs. Destabilizing mutations may therefore be tolerated or fixed through compensatory substitutions elsewhere in the protein or by contributing to functional innovation, particularly in regions under adaptive pressure (Tokuriki and Tawfik, 2009; DePristo et al., 2005).

However, the identification of only four codons (positions 93, 100, 163, and 177) under episodic positive selection (MEME, *p* ≤ 0.0396) suggests rare, site-specific adaptive events, potentially driven by antibiotic pressure and/or host-specific environments, rather than pervasive diversifying selection across the gene. These limited adaptive changes are consistent with the conserved architecture of the MsrA protein, in which the duplicated ATP-binding cassette nucleotide-binding domains (NBD1 and NBD2) and the inter-domain linker are functionally critical (Ross et al., 1990; Reynolds et al., 2003). In this context, three significantly conserved DNA regions (65–76 bp) were identified, exhibiting high sequence conservation (0.884–0.908) and homozygosity (H ≈ 0.975–0.980) across the *msrA* gene. These conserved regions map partially to the inter-domain linker (regions 1 and 2) and entirely to NBD2 (region 3), with no overlap in NBD1. Together, these patterns suggest that the structural integrity of the inter-domain linker and the NBD2 is particularly critical for MsrA-mediated ribosomal interactions and ATP-dependent conformational modulation, whereas NBD1 may tolerate slightly greater sequence variation. Overall, the evolutionary dynamics of the *msrA* gene reflect a balance between strong purifying selection maintaining core resistance functionality and rare episodic adaptations, likely facilitating persistence and spread in antibiotic-exposed niches without excessive allelic turnover. The moderate-to-high LD landscape (ZnS = 0.3600, Za = 0.4204, with long-range complete *D*′ = 1.0 persisting over >1 kb) suggests limited effective recombination in the sampled *msrA* backgrounds, such that recombination has been insufficient to fully erode linkage among polymorphisms. Consistent with this, the strong dominance of a few allelic variants—especially allele 19 in *S. aureus*—supports clonal expansion of epidemiologically successful lineages/allelic backgrounds as a major mode shaping *msrA* evolution within staphylococci.

### 4.2 Structured distribution of *msrA* alleles across plasmid mobility classes and implications for their dissemination

Here, we demonstrate that *msrA* allele distribution across plasmids is highly structured and non-random, with allele identity strongly associated with the plasmid mobility class. Specific alleles exhibited distinct mobility preferences, with alleles 10 and 8 enriched in conjugative plasmids, indicating preferential association with highly transmissible vectors, while alleles 17, 16, 2, and 14 were enriched in non-mobilizable plasmids, suggesting more constrained dissemination. Our findings are partially consistent with broader genomic evidence showing that antimicrobial resistance genes (ARGs) are preferentially associated with highly mobile plasmids (Coluzzi and Rocha, 2025) and that plasmid-mediated horizontal gene transfer (HGT) plays an important role in resistance dissemination among staphylococci (Schwarz et al., 2014). Importantly, our multivariable analysis revealed that allele identity remains an independent predictor of plasmid mobility after controlling for plasmid size and GC content. This indicates that allele–plasmid associations are not solely driven by general plasmid characteristics, such as size and GC content, but may reflect allele-specific compatibility with particular plasmid backbones. The previous studies have emphasized the role of plasmid structural features, including size and replicon type, in determining plasmid mobility (Zhang et al., 2023; Al-Trad et al., 2023). The present findings suggest that *msrA* allele identity profiles represent an additional layer of resolution for understanding the mobility patterns and trajectories of their carrier plasmids, at least within *Staphylococcus* and potentially beyond.

In our study, the role of *msrA*-carrying plasmid size in mobility appeared to be context-dependent. Although plasmid size did not differ significantly between mobility groups in unadjusted analyses, an association emerged after adjustment, largely driven by a small number of large conjugative plasmids. This observation is consistent with previous studies indicating that plasmid mobility is not strictly determined by size alone (Zhang et al., 2023; Coluzzi and Rocha, 2025). At the same time, other studies have reported that mobilizable plasmids are often smaller and more abundant (Al-Trad et al., 2023), suggesting that size may correlate with mobility in certain systems. Taken together, these findings indicate that plasmid size is not a primary determinant of the mobility of *msrA*-carrying plasmids and should therefore be interpreted in conjunction with other factors, such as gene content, mobility machinery, and plasmid background. A key insight from the plasmid analyses is that the dissemination of *msrA* alleles across species is strongly associated with plasmid background diversity. Alleles detected in multiple *Staphylococcus* species were carried by a greater number of plasmid clusters than species-restricted alleles (median: 3 vs. 1; *p* < 0.001), suggesting that the ability of *msrA* to occupy diverse plasmid backbones facilitates its interspecies spread. Furthermore, the repeated occurrence of identical allele–plasmid-cluster combinations across multiple *Staphylococcus* species provides strong evidence for plasmid-mediated interspecies transfer of *msrA*. These observations are consistent with established models of plasmid-driven gene flow within staphylococci and support the role of shared ecological niches in facilitating horizontal gene transfer (Schwarz et al., 2014).

### 4.3 *Staphylococcus* major species networks of *msrA* transmission

The detection of recombination signals (via GARD and RDP4) and the occurrence of broad-host alleles (e.g., allele 2, found across multiple *Staphylococcus* species and, rarely, in *Enterococcus* and *Corynebacterium*) indicate that HGT events contribute to the introduction of variation and facilitate the wider dissemination of *msrA*. The involvement of certain *Staphylococcus* allelic variants in intergeneric recombination events was further supported by ML phylogenetic inferences, strongly suggesting their transmission to *C. portucalensis,* as well as to a yet-unidentified species of *Pseudomonas*. *C. portucalensis* is a recently described species within the genus *Citrobacter*, which has been increasingly associated with emerging multidrug-resistant strains in clinical settings (Cao et al., 2021; Luo et al., 2022). It can be assumed that the above *Pseudomonas* strain acquired *msrA* from *S. aureus,* while the specific donor-species for this gene allelic variant (allelic type 2), carried by the *Streptococcus*, *Enterococcus* and *Corynebacterium* strains examined in this study, remain unclear. These findings are in line with the previous observation pinpointing the sharing of very high DNA identity (99–100%) of the specific *msrA* alleles among these organisms (Ojo et al., 2006). However, the broad distribution of this allelic variant across multiple *Staphylococcus* species, together with its closest phylogenetic relationships to the allelic types exclusively associated with the same genus, provides strong evidence for intergeneric recombination events, with *Staphylococcus* acting as a common donor of *msrA* to both *Enterococcus* and *Corynebacterium*.

The substantial plasmid bias, observed for the predominant *msrA* alleles (particularly allele 19), as well as the presence of specific allelic groups exclusive to these extrachromosomal elements, indicate that plasmids serve as the primary vehicles for *msrA* acquisition and horizontal transfer. Moreover, the alleles (2, 3, 16, 17, and 19) shared between the plasmids and chromosomes represent key “bridge” variants that likely sustain ongoing gene flow between plasmids and chromosomes within *Staphylococcus* spp. This pattern is consistent with broader staphylococcal genomics, in which plasmids and other mobile genetic elements (MGEs) frequently mediate the exchange of accessory genes, including those conferring resistance (Haaber et al., 2017; Firth et al., 2018; Liu et al., 2024). Future studies could prioritize the analyses of the flanking regions of these alleles to identify associated MGEs (e.g., insertion sequences, transposons, or integrases) and reconstruct specific transfer events. Such analyses would further clarify the mechanisms driving the observed allelic overlap and delineate the relative contributions of conjugation, transduction, and transformation to *msrA* dissemination across *Staphylococcus* and beyond.

The combined application of RDP4-implemented recombination detection algorithms alongside the split decomposition method coupled with the PHI test has proven effective in identifying networks of putative donors and recipients of antimicrobial resistance genes (ARGs) (Gabashvili et al., 2020; Tsiklauri et al., 2022; Gabashvili et al., 2022a, 2022b). This framework offers substantial advantages over the use of any single method, as the recombination detection algorithms embedded in RDP4 and SplitsTree interrogate distinct and complementary signals of HGT. Notably, RDP4 uniquely enables the inference of putative donor and recipient sequences (Martin et al., 2015), a capability not shared by other commonly used recombination detection software. In contrast, the split decomposition method identifies and visualizes recombination events as parallelogram structures reflecting parallel nucleotide substitutions with conflicting evolutionary histories (Bandelt and Dress, 1992), although it does not resolve the trajectories of these events. The PHI test further strengthens this analytical framework by detecting significant site incompatibilities via quantitative assessment of homoplasy, providing statistical support for recombination-driven parallel substitutions rather than strictly clonal or convergent evolutionary processes (Bruen et al., 2006).

Based on the inferences derived from the combined RDP4 and SplitsTree analyses, we propose that *S. aureus* has acted as one of the major donors mediating the dissemination of *msrA* loci across the *Staphylococcus* genus. In particular, *S. aureus* appears to have served as the major donor of *msrA* to *S. chromogenes* and *S. saprophyticus*. Conversely, reciprocal transfer events inferred by RDP4 positioned *S. epidermidis*, *S. equorum*, *S. haemolyticus*, *S. hominis*, *S. cohnii*, and even *C. portucalensis* as the major donors of *msrA* to *S. aureus*, *S. chromogenes*, and *S. saprophyticus*, collectively exhibiting both interspecies and intergeneric recombination involving this gene. Besides, the SplitsTree-inferred parallelograms supported by the highest fit values (100), strong bootstrap support (≥99.3), and the highly significant PHI test *p*-values (≤0.0018) provide additional compelling evidence for interspecies recombination events within *Staphylococcus*. In contrast, the additional parallelograms associated with specific parallel nucleotide substitutions, although supported by strong fit and bootstrap values, were not corroborated by PHI test statistics, and may instead reflect convergent evolution acting on *msrA*.

Taken together, these recombination signals, combined with the distribution, genetic relatedness, and sharing of *msrA* allelic variants, position *S. aureus* populations as at least one of the central hubs within the donor–recipient networks governing *msrA* dissemination across the *Staphylococcus* genus. The predominance of plasmid-associated alleles (e.g., 80% for allele 19 and 76% for allele 2) underscores the critical role of plasmids as vectors facilitating rapid intra- and interspecies transfer of *msrA* in environments harboring mixed staphylococcal populations. Moreover, the frequent detection of plasmid-borne *msrA* alleles within the staphylococcal chromosomes highlights the role of plasmids as transient vehicles that seed this resistance determinant into the more stable chromosomal gene pool. The observed pattern is consistent with a classic signature of adaptive evolution driven by MGE-induced trafficking of ARGs, a phenomenon well documented in staphylococci (Schwarz et al., 2014; Firth et al., 2018). The plasmid-mediated horizontal transfer of *msrA,* coupled with its chromosomal integration, poses a significant clinical threat, as it facilitates the rapid dissemination and durable persistence of macrolide- and type B streptogramin-resistance across staphylococci.

While this study incorporates an almost comprehensive set of the *msrA* nucleotide sequences currently available in the NCBI GenBank database, certain limitations and potential biases inherent to publicly available datasets should be acknowledged. These may include clinical sampling bias, geographic underrepresentation or overrepresentation, sequencing project–specific biases, differential availability of plasmid versus chromosomal sequences, and database enrichment of epidemic clones carrying *msrA*. It should also be acknowledged that both the uneven distribution of DNA sequence data on *msrA*-carrying plasmids and the diversity of alleles of this gene among coagulase-negative staphylococci in microbial genome databases represent another limitation of this study. Such factors could influence the observed allele frequencies, linkage patterns, and inferred transmission dynamics.

## 5 Conclusion

This study presents a comprehensive evolutionary and molecular epidemiological framework for elucidating the persistence and dissemination of the macrolide- and streptogramin B–resistance gene *msrA* in *Staphylococcus*. While *msrA* evolution is predominantly constrained by strong purifying selection preserving its essential function, episodic diversifying selection at specific sites enables localized adaptive changes—sometimes involving structurally destabilizing substitutions—that may enhance functional flexibility under antibiotic or host-driven pressures. The high conservation of the inter-domain linker and nucleotide-binding domain 2 further underscores their essential role in maintaining resistance function. Plasmid-mediated horizontal gene transfer appears to be one of the primary mechanisms underlying *msrA* dissemination across staphylococci. The ability of certain alleles of this gene to occupy diverse plasmid lineages emerges as one of the important “drivers” of interspecies spread of *msrA* within *Staphylococcus.* A restricted subset of “bridge” alleles facilitates gene flow between chromosomal and plasmid locations and across species boundaries within *Staphylococcus* and beyond. In this network, *S. aureus* acts as a central hub, promoting both interspecies transmission within the genus and occasional intergeneric transfer to taxa such as *Enterococcus*, *Corynebacterium*, *Pseudomonas*, and *Citrobacter*. Collectively, these patterns identify staphylococcal populations—particularly *S. aureus*—as key reservoirs and amplifiers of resistance to macrolide and streptogramin B. Overall, our findings support a dual evolutionary strategy underlying the success of *msrA*: the long-term preservation of essential resistance function of this gene through purifying selection, coupled with its efficient dissemination via mobile genetic elements and clonal expansion. This combination enables *msrA* to persist and become entrenched across diverse bacterial communities within *Staphylococcus* and beyond. These findings highlight the clinical importance of monitoring plasmid-borne *msrA*, particularly within *Staphylococcus aureus*, as a key reservoir driving resistance dissemination, and support the integration of allele-level and plasmid-context information into surveillance strategies. Future studies combining genomic and functional approaches will be essential to resolve transmission dynamics and inform targeted interventions to limit the spread of macrolide and streptogramin B resistance.

## Data Availability

The DNA sequence data analyzed in this study are publicly available in the NCBI GenBank database (https://www.ncbi.nlm.nih.gov/) under their respective accession numbers, listed in the article Supplementary material (see Supplementary file “Table 1.xlsx”, sheet “Table S1”).

## Footnotes

1
https://www.ncbi.nlm.nih.gov/

2
https://card.mcmaster.ca/home

3
https://www.uniprot.org/id-mapping

4
https://itol.embl.de/
