# Supplementary material for: Evolution and transmission landscape of the staphylococcal msrA gene mediating resistance to 14-membered macrolides and type B streptogramins
Source: Front Microbiol. 2026 Apr 22;17:1815688. doi: 10.3389/fmicb.2026.1815688 (PMC13144154; doi:10.3389/fmicb.2026.1815688)
Supplement: Supplementary file 2 [file Image_1.pdf]

Supplementary Material

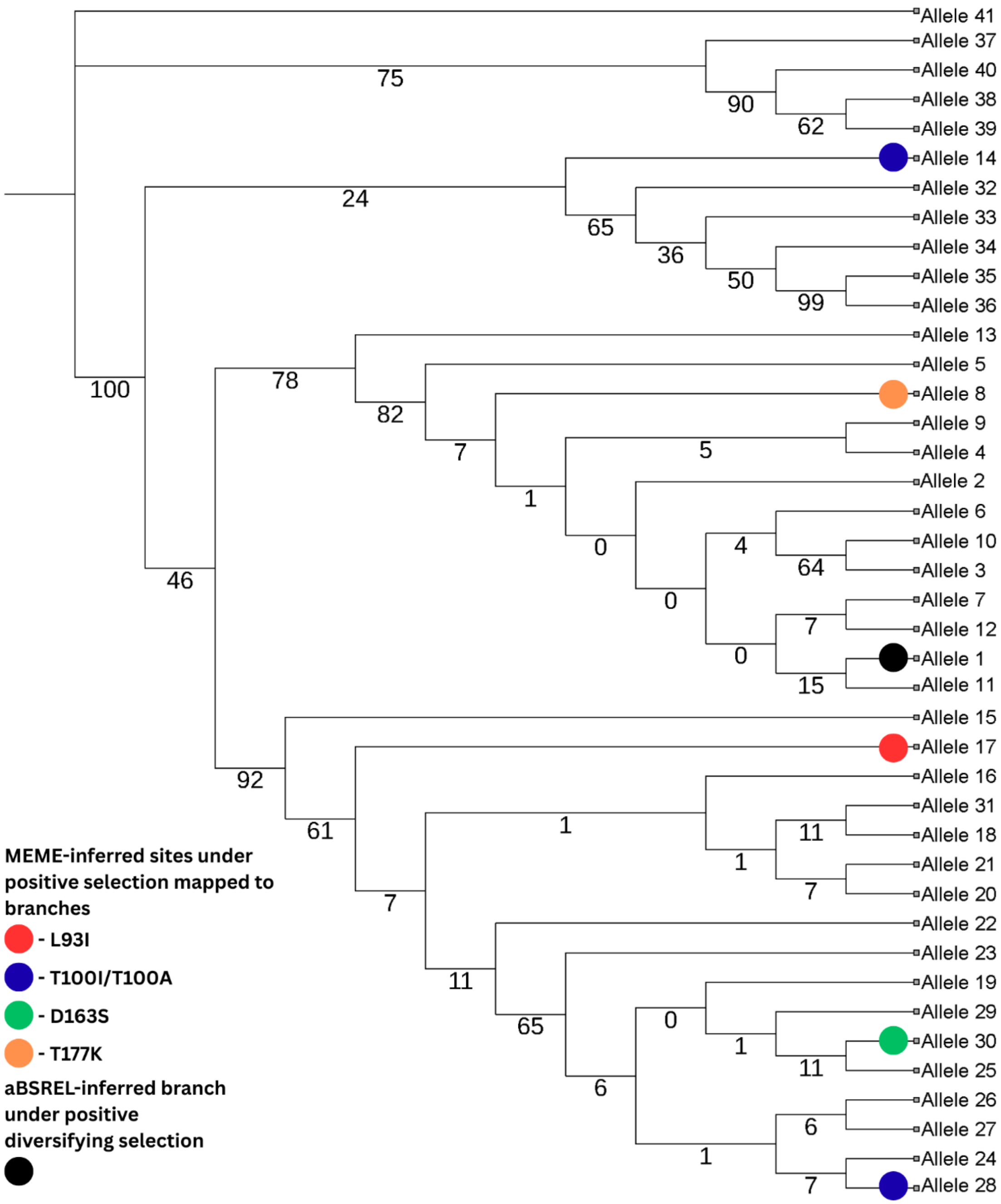

Supplementary Figure S1. The ML tree elucidating the genetic relationships among distinct allelic variants of the *msrA* gene.

The ML tree was reconstructed using IQ-TREE 2 (v3.0.1) (Minh et al., 2020), with ModelFinder (MFP) selecting MG+F3X4+G4 as the best-fit substitution model under the Bayesian Information Criterion (BIC).
